# Health of Black children and youth in Canada: a scoping review

**DOI:** 10.1186/s12889-025-24474-6

**Published:** 2025-09-02

**Authors:** Chiedozie James Alumona, Aloysius Nwabugo Maduforo, Oluwagbohunmi Adetunji Awosoga, Nicole A. Johnson, Shannon D. Scott, Caitlin McClurg, Dominic A. Alaazi, Bukola Salami

**Affiliations:** 1 Department of Community Health Sciences, Cumming School of Medicine, University of Calgary, Calgary, AB Canada; 2 Faculty of Health Sciences, University of Lethbridge, Lethbridge, AB Canada; 3 Department of Pediatrics, University of Calgary, Calgary, AB Canada; 4 Faculty of Nursing, University of Alberta, Edmonton, AB Canada; 5 Libraries and Cultural Resources, University of Calgary, Calgary, AB Canada; 6 School of Health Studies, Faculty of Health Sciences, Western University, London, ON Canada

**Keywords:** Canadian African immigrants, African, Caribbean, Social determinant of health, Health inequity, Racism

## Abstract

**Background:**

Black children and youth in Canada experience significant health inequity due to systemic racism, socioeconomic inequities, and inadequate access to culturally responsive healthcare services, affecting their overall well-being. This scoping review mapped and synthesised empirical evidence on the health of Black children and youth in Canada to inform policy and practice.

**Methods:**

The review followed Arksey and O’Malley’s scoping review framework and adhered to the Preferred Reporting Items for Systematic Reviews and Meta-Analyses extension for Scoping Reviews (PRISMA-ScR) guidelines. Seven databases (Ovid MEDLINE, EMBASE, APA PsycINFO, Web of Science, Scopus, Academic Search Complete, and SocINDEX) were searched for empirical studies published in peer-reviewed journals. Studies were included if they focused on Black children and youth (0–30 years) residing in Canada and their findings were synthesised thematically.

**Results:**

A total of 56 health-related studies were included. The included studies were conducted across all the Canadian provinces and territories between 1993 and 2024, with 87.5% based in a single province and 48.2% published between 2021 and 2024. Quantitative studies accounted for 55.4% of the total. The health conditions identified included sickle cell disease, preterm birth, HIV, pediatric lupus, and mental health disorders such as depression, PTSD, and psychosomatic symptoms (reported in up to 81.7% of participants in one study). Racism was a prominent social determinant contributing to health conditions and barriers to healthcare access. Cultural practices and religiosity protected the Blacks from engaging in risky lifestyles while contributing to the distorted view of some illnesses.

**Conclusion:**

A high variety of health conditions were identified, with racism being a key determinant of health for Black children and youth in Canada. Culturally responsive, anti-racist health policies, community-led health education, and equitable access to services are critical to improving health outcomes.

**Supplementary Information:**

The online version contains supplementary material available at 10.1186/s12889-025-24474-6.

## Background

Health is shaped by a complex interplay of biological, social, and environmental factors. Among children and youth, early life experiences, access to resources, and social conditions significantly influence their health trajectories [1]. For Black children and youth in Canada, these pathways are further influenced by systemic inequities, particularly racism, discrimination, and socioeconomic marginalisation, that create unique and intersecting challenges to their health and well-being. Improving the health of this population aligns with international commitments outlined in the United Nations Sustainable Development Goals (SDGs), particularly SDG 3 (Good Health and Well-being) and SDG 10 (Reduced Inequalities), which emphasise the importance of addressing health inequity and systemic barriers among marginalised groups [2].

Despite Canada’s policy commitment to equity and inclusion, a growing body of evidence underscores persistent health inequity experienced by Black populations across various domains, including mental health, healthcare access, maternal and child health, and chronic disease management [3–5]. However, these studies often address Black populations as a homogenous group, with limited attention to age-specific experiences or developmental stages, leaving critical gaps in our understanding of how the health of Black children and youth is affected. The demographic significance of this population adds urgency to this inquiry. In 2021, Black Canadians comprised approximately 1.5 million individuals, more than double the population recorded in 1996, with nearly one-third under the age of 25 [6, 7]. This growth reflects an increasingly diverse and dynamic community and highlights the importance of incorporating Black children and youth’s voices and experiences into health research and policymaking.

The concept of social determinants of health (SDOH), as outlined by the World Health Organisation (WHO), emphasises the conditions in which people are born, grow, live, work, and age, and their profound influence on health inequities [8]. However, it is not solely the existence of these conditions that drives health inequities; rather, it is their unequal distribution, shaped by systemic structures of power, discrimination, and entrenched bias, that sustains and exacerbates health inequities across populations. In Canada, Black children and youth are disproportionately affected by the disparities in housing, education, income, and exposure to systemic racism. For instance, in 2016, Black women were 27% less likely to complete high school and 21% less likely to complete university than their White counterparts. Additionally, only 1.8% of Canadian elementary and secondary school teachers were Black, and 33% of Black children lived in low-income households compared to 12.7% of White children [3, 4]. These social disparities and structural power play translate into unequal health outcomes. For example, compared to their White counterparts, Black individuals report higher rates of chronic conditions such as diabetes and lower levels of physical activity [3, 4].

To date, no scoping or systematic review has focused exclusively on synthesising empirical evidence related to the health and SDOH of Black children and youth in Canada. Existing reviews tend to aggregate data across racialised groups or adult populations, failing to capture the specific and nuanced experiences of Black youth. Therefore, this scoping review aimed to map existing empirical evidence on the general health of Black children and youth in Canada, highlight the SDOH, and offer actionable insights for policymakers, healthcare providers, and community stakeholders.

## Methods

The scoping review was conducted using the framework developed by Arksey and O’Malley [9]. This framework consists of a five-stage methodology: identifying the research question, identifying and selecting relevant studies, charting the data, and collating, summarising, and reporting the results. The scoping review was reported following the Preferred Reporting Items for Systematic Reviews and Meta-Analyses extension for Scoping Reviews (PRISMA-ScR) checklist [10], which is given in Supplementary File 1.

### Protocol

The protocol was developed by content experts in the well-being of Black children or youths and methodological experts in the scoping review (BS, DAA, ANM & CM). It covered all studies related to Black children and youths. During the title and abstract screening, we included articles on these five thematic areas: health, education, child welfare, criminal justice, and migration and settlement. However, due to the large number of published studies for each thematic area, the research team decided to publish them separately. Therefore, the search strategy for the whole study was described, but this manuscript focused on the health thematic area.

### Eligibility criteria

Studies were included if (1) they were peer-reviewed empirical research of quantitative, qualitative, and mixed designs published in journals (2) involved Black (Canadian-born, African and Caribbean descent) children and youth (0–30 years) residing in Canada, (3) limited to Canada, (4) written in either English or French Language, and (5) included one of the thematic areas (health, education, child welfare, criminal justice, and migration and settlement). The decision to include individuals up to age 30 was informed by culturally relevant understandings of youth within African and Caribbean communities, where the transition to adulthood is shaped by intersecting structural, cultural, and socioeconomic factors. This broader age range also reflects definitions used in Canadian health and social policy frameworks that address the experiences of Black and racialised youth, where extended transitions to adulthood are often linked to systemic barriers such as employment discrimination, limited access to services, and economic precarity [11]. The exclusion criteria were studies that focused exclusively on adults, those not relevant to the identified thematic areas (this was identified at the title and abstract screening stage, but all studies on Black people were searched and imported into Covidence), or research conducted outside Canada. Additionally, systematic reviews, narrative reviews, scoping reviews, and non-peer-reviewed studies were excluded.

### Information sources

Following the Bramer and colleagues’ [12] recommendation of combining databases, we searched multiple databases to ensure a thorough review of literature in diverse fields, including health, social sciences, psychology, and community studies. The following databases were searched on 20 May 2024, starting from the year they were established: Ovid MEDLINE, EMBASE, APA PsycINFO, Web of Science, Scopus, Academic Search Complete, and SocINDEX. Although PubMed was not searched separately, its indexed content was comprehensively captured through our search of Ovid MEDLINE.

### Search strategy

Search terms were identified through consultations among the content experts in the team and the librarian. The search strategy was developed and adapted for all databases (Supplementary File 2). The search was structured around three main components: geographical scope (Canada and its provinces and territories), population (Black [African and Caribbean descent]), and age group (children and youths). When possible, subject headings from controlled vocabularies (e.g., MeSH) were used in the search. The search sensitivity was enhanced by entering concepts in the search string as keywords with truncation (*) and explode (exp) when appropriate. Boolean operators (AND, OR) connected subject headings and keywords.

### Selection of sources of evidence

**Fig. 1:**
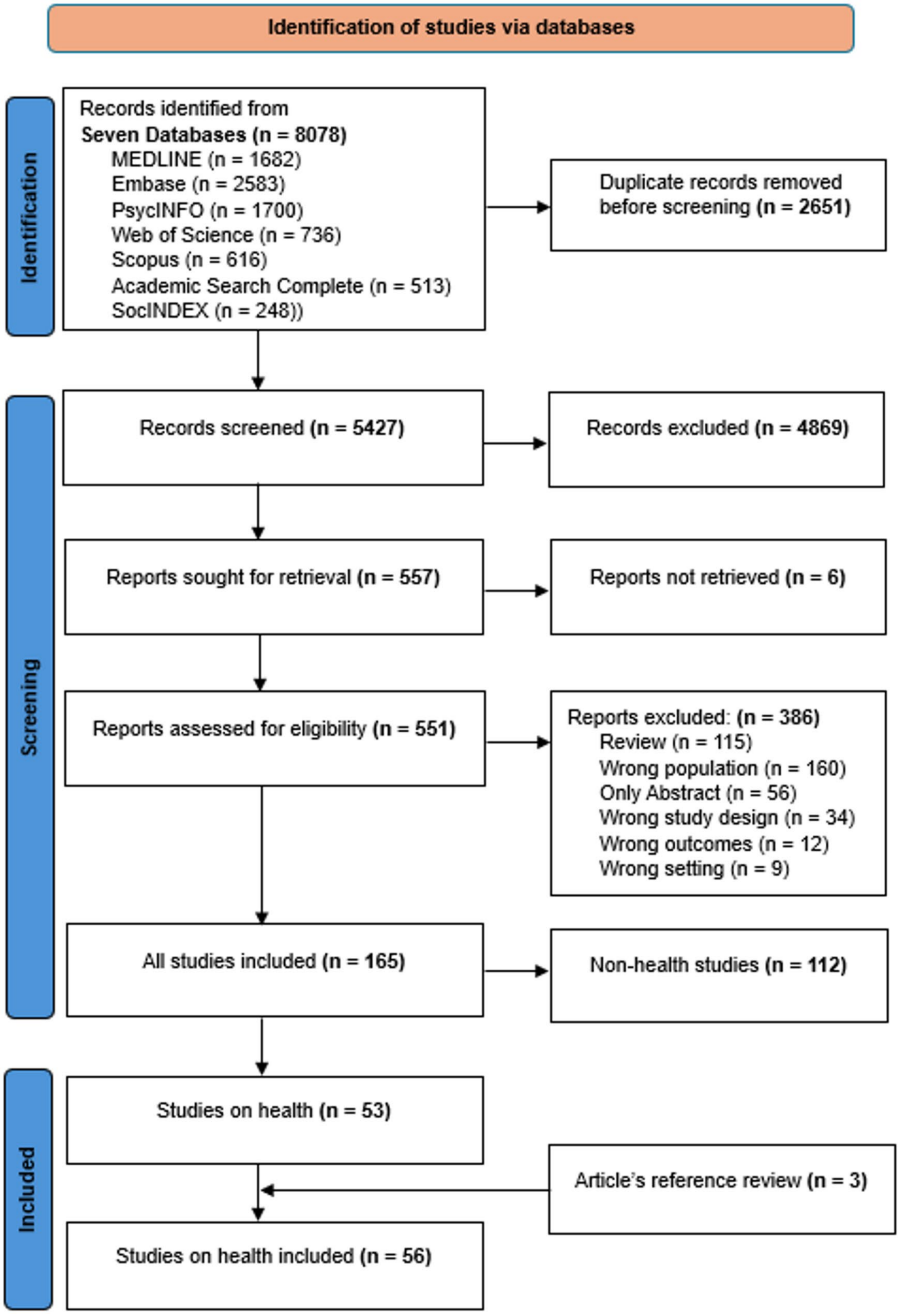
PRISMA flowchart

The retrieved citations from all the databases were exported to Covidence, a web-based systematic review management software, for title and abstract screening and full-text screening. Trained research assistants were involved in the screening process, and we conducted pilot screening at each stage to ensure inter-reviewer agreement ahead of the main screening. The Covidence randomly assigned articles to two independent screeners and conflict resolution to a third independent reviewer (BS, DAA and ANM). Additional articles were found by hand-searching the references of the included articles. See Fig. 1 for the details of all included articles.

### Data charting/extraction process

The included articles were first organised into five thematic areas: health, education, child welfare, criminal justice, and migration and settlement. A trained research assistant conducted the initial data extraction for articles within the health theme using a standardised Microsoft Excel spreadsheet. This extracted data was reviewed for accuracy by one author (CJA), with additional input from two co-authors (ANM and BS) to ensure consistency in interpretation. Prior to full data extraction, a pilot exercise was conducted on a small subset of articles to establish inter-reviewer agreement and refine the extraction framework. All authors subsequently reviewed the extracted data across themes during the synthesis stage to validate the findings and ensure comprehensive coverage of the literature. Details of the extracted variables and data charting framework are provided in Supplementary File 3.

### Data items

The following information was extracted from each article: authors, title, year of publication, province or territory of study, study design, data collection source (e.g., Black children and youth, parents, service providers), sample size, child and youth age range/mean, gender, country/region of origin of participants, data collection methods (e.g., interviews, surveys, focus groups), and key findings.

### Synthesis of results

Descriptive statistics of frequency and percentage were used to summarise the study characteristics, including the year of publication, provinces of study, study design, data collection source, and gender. A narrative synthesis was conducted by grouping the findings of the included studies into five themes: health conditions, identity-based discrimination, social determinants of health, healthcare service utilisation, and lifestyle. Quality appraisal of the included studies was not completed, as scoping reviews aim to offer an overview or map of the pertinent evidence [13].

## Results

A total of 8078 citations were retrieved across the seven databases, of which 2651 duplicates were removed. The PRISMA flowchart (Fig. 1) shows that 5427 citations went through the screening process, excluding 4869 articles that did not meet eligibility criteria. We could not retrieve six of the remaining 557 articles through the library’s collection, leading to the full-text screening of 551 articles. The full-text screening yielded 165 articles for extraction, of which 53 were health-related. Three additional health-related articles were found through a review of the references of the included articles, yielding a total of 56 articles.

**Fig. 2:**
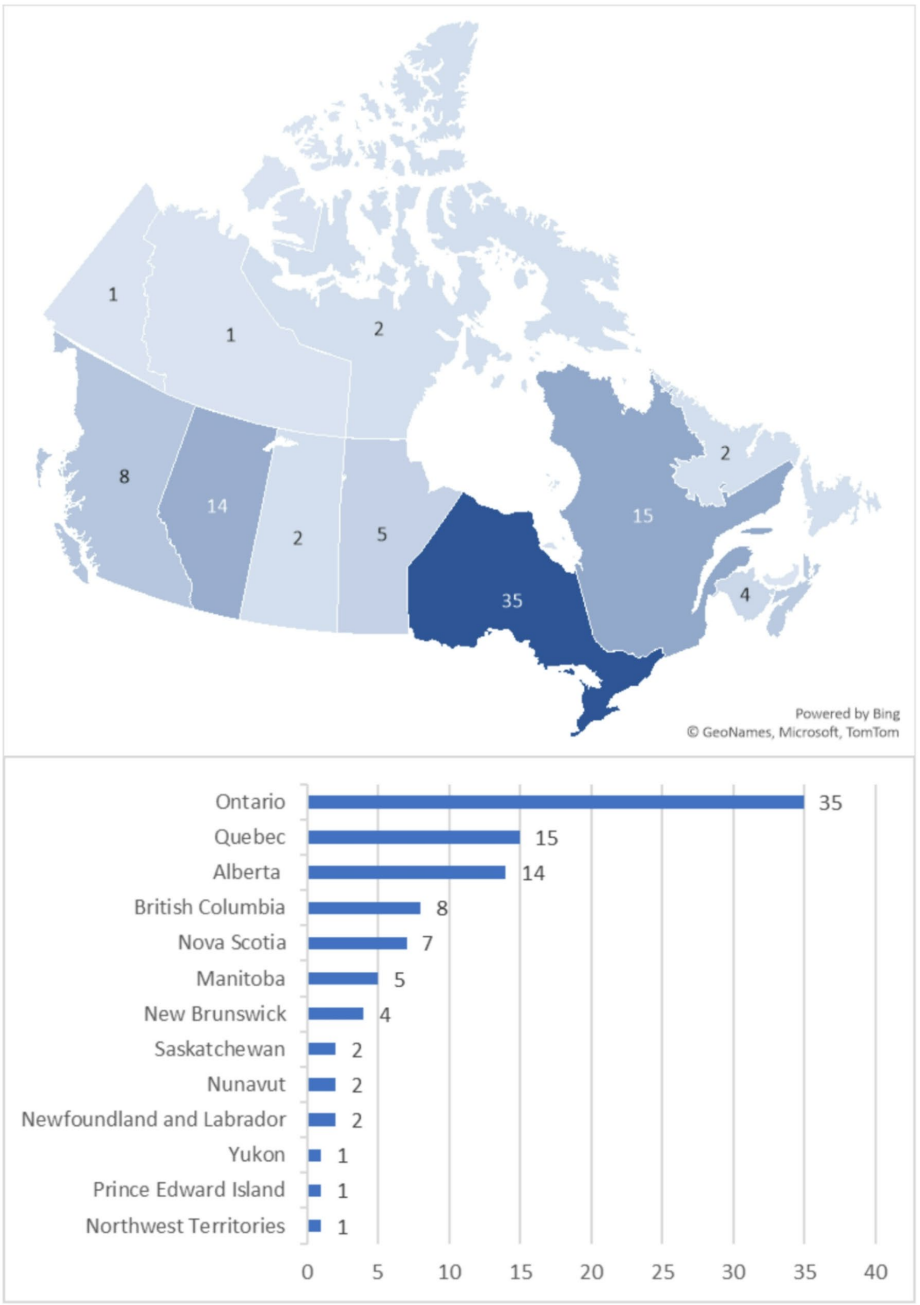
Provinces where included studies were conducted

The studies were conducted across all Canadian provinces and territories (Fig. 2) between 1993 and 2024. Table 1 shows that most studies were conducted in a single province (*n* = 49, 87.5%) between 2021 and 2024 (*n* = 27, 48.2%). Many of the studies employed a quantitative design (*n* = 31, 55.4%) and collected data from youth only (*n* = 35, 62.5%), including both males and females (*n* = 38, 67.9%) of African descent (*n* = 18, 32.1%). The sample sizes ranged from 8 to 2,124,909.

**Table 1:** Study characteristics

Characteristics	Frequency	Percentage
**Number of Provinces/territories**		
Single	49	87.5
Multiple	7	12.5
**Year of publication**		
1993–2000	3	5.4
2001–2020	26	46.4
2021–2024	27	48.2
**Study design**		
Qualitative	22	39.3
Quantitative	31	55.4
Mixed methods	3	5.4
**Data collection source**		
Children only	9	16.1
Youth only	35	62.5
Parents only	5	8.9
Professionals only	2	3.6
Combined sources	5	8.9
**Gender**		
Males only	3	5.4
Females only	4	7.1
Transgender/non-binary only	3	5.4
Males and females	38	67.9
Males, females, and others	6	10.7
Not stated	2	3.5
**Participants’ origin**		
African	18	32.1
Caribbean	4	7.2
Both African and Caribbean	18	32.1
Not stated	16	28.6

### Synthesis of results

#### Health conditions

The health conditions reported among Black (African and Caribbean) children and youth in Canada included sickle cell disease [14–17], adverse birth outcomes such as preterm birth, low birth weight, and small-for-gestational-age [18, 19], Vitamin D deficiency [20], mental/emotional problems [21–24] such as post-traumatic stress disorder (PTSD), psychosis, depression, anxiety, and stress [25–29], Human immunodeficiency virus (HIV) [30], malaria [31], pediatric systemic lupus erythematosus [32], psychosocial problems such as conduct disorder and problem behaviour [33, 34], and psychosomatic symptoms [35]. Two studies reported on poor self-rated health outcomes and explored suicidal thoughts and attempts [36, 37]. Table 2 shows the prevalence of health conditions.

**Table 2:** Prevalence of health conditions

Health conditions	Prevalence	Author & year	Study design
Sickle cell disease	Not reported	Aiko Bruce et al., 2018	Qualitative
	Not reported	Monagel et al., 2022	Quantitative
	15.4% of infants had sickle cell disease or trait	Robitaille et al., 2006	Quantitative
	Back pain among children with sickle cell disease was 26%	Roger & Letts, 1999	Quantitative
Adverse birth outcomes	8.9% of infants born to Black mothers were preterm.	McKinnon et al., 2016	Quantitative
	Haitian-born mothers: preterm birth (8.5%), low birth weight (7.3%), and small-for-gestational-age (12.6%). Canadian-born mothers: preterm birth (5.8%), low birth weight (4.9%), and small-for-gestational-age (11.5%).	Auger et al., 2012	Quantitative
Vitamin D deficiency	Vitamin D deficient (31%) or insufficient (37%).	Grégoire-Pelchat et al., 2018	Quantitative
Mental/emotional problems	Not reported	Osman et al., 2024	Qualitative
	Not reported	Olawo et al., 2021	Qualitative
	Not reported	Salami, Alaazi, et al., 2022	Qualitative
	Not reported	Salami, Idi, et al., 2022	Qualitative
	Not reported	Beiser et al., 2012	Quantitative
	67.11% of participants reported probable post-traumatic stress disorder	Cénat, Dalexis, et al., 2023	Quantitative
	40.94%, 44.50%, and 31.36% of participants had clinically meaningful anxiety, depression, and stress levels, respectively.	Cénat, Farahi, et al., 2023	Quantitative
	65.87% of participants reported severe depressive symptoms	Cénat et al., 2021	Quantitative
	Not reported	van der Ven et al., 2012	Quantitative
Human immunodeficiency virus (HIV)	1.3% of Montrealers of Haitian origin have HIV	Adrien et al., 1999	Quantitative
Malaria	73% had had at least 1 episode of malaria	Ndao et al., 2005	Quantitative
Pediatric systemic lupus erythematosus (pSLE)	Proportion of Blacks with pSLE that had renal disease was 64%	Hiraki et al., 2009	Quantitative
Psychosocial problems	Not reported	Okoye et al., 2023	Quantitative
	Conduct disorder diagnosis was 12.7%.	Rousseau et al., 2008	Quantitative
Psychosomatic symptoms	81.7% of participants experienced psychosomatic symptoms	Cénat et al., 2022	Quantitative
Suicidal thoughts and attempts and Poor health	Not reported	Matheson et al., 2008	Quantitative
	Not reported	Okoye & Saewyc 2021	Quantitative

### Identity-based discrimination

#### Gender

Gender power dynamics were reported to influence sexual activities/health, often preventing women from negotiating sexual encounters or discussing sexual matters with their partners [38]. Being a female was associated with higher levels of psychosomatic symptoms [35]. Restrictive social norms limited women’s sexual health experiences, hindering the expression of their sexual desires [39].

Conversely, men often perceived masculinity as the ability to control their sexual power and to provide for, protect, love, and lead their families [40]. The trauma of systemic oppression altered traditional notions of masculinity, requiring men to deconstruct this trauma-altered identity [41].

#### Race

Racism was identified as a significant predictor of depressive symptoms [26]. It was positively associated with anxiety [25], PTSD symptoms [27], suicidal thoughts and attempts, poor health, and extreme stress [36], psychosomatic symptoms [35], increased HIV risk [42], and psychosocial problems [33]. It was reported to contribute to mental health problems [21, 22]; and its effect was compounded by the COVID-19 pandemic [24].

### Social determinants of health

#### Income

Poverty was reported to increase the risk of HIV [42] and hinder children’s physical activity experiences [43].

#### Socio-cultural factors

Sociocultural factors, including religion, culture, and family, were reported to contribute to negative attitudes toward homosexuality [44]. On the other hand, community resilience, characterised by trust, faith, strengths, and values, was found to be negatively associated with anxiety, depression, and stress [25].

The reported negative impacts of sociocultural factors included a distorted perception of AIDS as a disease of the devil [45] and mental health issues as madness or spiritual problems [46]. Religion was also reported to contribute to mental health problems [22]. Moreover, there was often a disconnect between families and healthcare providers regarding the understanding of diabetes, negatively impacting diabetes management [47]. Parent-child communication about sexual health was less likely to occur unless prompted by media or peers [48, 49]. The family breakdown and absence of family or community discussions about sex were reported to increase the risk of HIV [42].

#### Socio-environmental factors

Living close to enabling environments was reported to enhance children’s physical activity experiences, while weather constituted a barrier [43]. Kerr et al. [50] reported that disadvantaged neighbourhoods had higher HIV stigmatising beliefs. Community leaders within the Black community identified that a lack of community services could increase the risk of HIV [42].

### Healthcare service utilisation

The utilised healthcare services in the literature included the COVID-19 vaccine [51, 52], HIV testing and care [53, 54], dental care [55], mental health services [56] such as psychosis and psychiatry care [57–59], blood donation [60], and primary health care physician [59].

The factors facilitating access to these services were health literacy and personal relationships [51, 52]. In contrast, the barriers to utilising these services were numerous, including a lack of trust in care providers, insufficient knowledge about illness and existing services, poverty, lack of Black service providers, and feeling dismissed by healthcare providers [42, 51–53, 55–57, 60]. The most frequently reported barriers were stigma, discrimination, and racism [36, 51, 52, 54, 56, 57, 60]. There were differences in access to these services among groups, with some having more opportunities to obtain care than others [58], resulting in a lower utilisation prevalence [59].

### Lifestyle

The disease-preventive practices of Black African and Caribbean children and youths reported in literature included seeking health knowledge and medical advice [61, 62], using a condom during sexual intercourse [62, 63], and having various sources of sexual health information [64]. However, the reported risky lifestyles were early sexual activities and experiences [65, 66], cigarette smoking [36], and substance use [67], including opioid [68].

Retaining protective cultural practices and participating in religious activities were reported to influence the population to avoid risky sexual activities [44]. However, discrimination [67] and incarceration [68] were reported to impact them negatively, leading to death [68]. Kengneson et al. [69] reported that mothers expressed concerns about their children’s obesity, which could have resulted from poor diet quality and/or stress.

## Discussion

Black people in Canada encounter multifaceted challenges that impact their health and well-being. Therefore, we reviewed the existing literature on the health of Black (African and Caribbean) children and youth in Canada, focusing on their health conditions, identity-based discrimination, SDOH, use of healthcare services, and lifestyle choices. Our findings reveal a high number of health conditions were identified where racism was noted as a negative influence. Black children and youth tend to engage in both health-promoting and risky lifestyles. Factors such as discrimination and incarceration influenced some people to adopt risky lifestyles, while cultural and religious activities served as protective mechanisms for others. These sociocultural influences also contributed to a negative perception of illness. Additionally, socioeconomic issues like poverty further increased disease risk and hampered access to healthcare. The most reported barriers to accessing healthcare services included stigma, discrimination, and racism, resulting in lower rates of service utilisation.

Racism is recognised as a significant determinant of health, contributing to ethnic inequities or differences in health [70, 71]. Our findings highlight the negative effects of discrimination and racism on health outcomes, utilisation of services, and lifestyle choices. Experiences of racial discrimination can be traumatising and can trigger stress responses and activate the hypothalamic-pituitary-adrenal axis, resulting in dysregulated cortisol production [72, 73]. This biological response to racial discrimination has been linked to poorer physical and mental health outcomes [74]. Racial discrimination has also been associated with disturbances in neural processes, with changes noted in brain areas, including the anterior cingulate cortex and prefrontal cortex, leading to poor mental health [75]. Previous systematic reviews have reported the negative impacts of racial discrimination on the health outcomes of children and youth [76, 77].

Racism in the healthcare system and the implicit bias of the health care providers lead to mistrust, reducing the likelihood of seeking timely medical care among Blacks [78, 79]. Even the underrepresentation of Black service providers is rooted in systemic racism [80], which starts in the education system [81], contributing to lower educational attainment among Blacks and fewer medical school enrolments. Those who enrol and become healthcare providers often report experiencing racism in their educational institutions and workplaces [82–84].

Risky lifestyle choices may be a coping mechanism for the psychological effects of perceived racial discrimination and experiences of incarceration [68, 85]. Imprisonment and exposure to a discriminatory environment can lead to feelings of worthlessness and hopelessness [86], resulting in increased engagement in risky behaviours and creating another pathway that links racial discrimination to poor health outcomes. Furthermore, the stereotyping of healthy cultural/religious practices of Black individuals during interactions with their peers can result in the adoption of risky lifestyles [85, 87].

While the Black community’s sociocultural activities fostered protective behaviours against risky lifestyles, their limited access to accurate, culturally responsive health information and differing perceptions of illness may have contributed to an increased risk of certain diseases. Many individuals tend to spiritualise illness and prefer alternative healing methods over appropriate medical care, which can lead to prolonged suffering and delayed diagnosis [45, 46]. Additionally, certain illnesses, particularly mental health issues, could be underreported due to the existing stigma surrounding them within the Black community [88].

These negative perceptions, often reinforced by financial barriers and the challenges of living in disadvantaged neighbourhoods due to systemic inequities, could constitute barriers to health and to accessing healthcare services [42]. When these factors intersect with sexism, female Black individuals may be disproportionately affected due to their traditional gendered responsibilities, such as childbearing, childcare, and other forms of unrecognised labour [89].

### Strengths and limitations

A major strength of this scoping review lies in its broad and inclusive scope, capturing multiple dimensions of the health of Black children and youth in Canada. The search strategy was systematically developed and implemented across multiple databases, beginning from their inception dates to ensure comprehensive coverage and reduce the risk of omitting relevant studies. Methodological rigour was upheld through pilot testing of the screening protocol, dual independent screening by trained research assistants, and conflict resolution by a third reviewer. Data extraction was conducted by one co-author (CJA), reviewed for accuracy and interpretive consistency by two others (ANM and BS), and validated by the full team during synthesis. Additionally, the five analytical themes used to organise the findings were developed inductively through an iterative and collaborative process, enhancing the conceptual depth and coherence of the synthesis.

The geographic distribution of the studies revealed a gap in coverage from certain regions, with few studies originating from Saskatchewan, Newfoundland and Labrador, Prince Edward Island, and the Yukon, Nunavut, and Northwest Territories. This may limit the generalisability of findings to underrepresented jurisdictions. Furthermore, none of the included studies focused specifically on how intersecting social determinants, such as race, gender, income, and geographic disadvantage, jointly affect the health outcomes of marginalised subgroups (e.g., Black female youth living in low-income neighbourhoods). Additionally, the included studies employed an observational design, limiting the potential to draw causal inferences. Moreover, this review did not aim to assess causality or the strength of associations between variables, and all interpretations should be understood within that context.

Although we adopted an inclusive age range of 0 to 30 years to reflect culturally and policy-relevant definitions of youth, particularly within African and Caribbean communities, the findings were not stratified by developmental stage (e.g., children vs. youth) due to inconsistent age reporting across studies and the thematic focus of the review. Additionally, the review included only peer-reviewed empirical literature; grey literature was excluded due to feasibility constraints, which may have introduced publication bias and excluded valuable insights from community or policy contexts. While formal quality appraisal was not conducted, in accordance with scoping review methodologies [9], we acknowledge that some included studies were limited by small sample sizes and a lack of longitudinal data. Finally, although great care was taken in generating themes inductively, the interpretive process may still be influenced by reviewer subjectivity due to the heterogeneity of the included literature.

### Implications for policy, practice, and research

This scoping review identified significant health conditions among Black children and youth in Canada, all affected and, in some cases, amplified in parallel by systemic racism and the social determinants of health. These findings necessitate immediate, multisectoral collaboration among policymakers, healthcare providers, researchers, and community stakeholders. Anti-racism frameworks that support systemic reforms across healthcare, education, and social services must be developed. The frameworks should include cultural competence training for providers, active counter-implicit bias, and workforce diversification to improve trust and care accessibility for Black individuals.

We recommend policymakers address the systemic barriers through targeted investments in affordable housing, equitable education, food security, and economic empowerment programs. Healthcare systems should adopt culturally responsive practices and increase the representation of Black professionals and formally integrating Black-led community organisations into service delivery. Evidence-based models, such as TAIBU Community Health Centre’s *Afya Program* and Youth Wellness Hubs Ontario, demonstrate the efficacy of culturally grounded, integrated care. Scaling such initiatives requires dedicated funding and institutional commitment to community partnerships.

To address cultural barriers to care, practitioners can implement community-based participatory services, co-design health promotion initiatives with Black youth and families, and collaborate with faith leaders and cultural mentors. Black healthcare professionals can also lead public health campaigns to ensure messaging resonates with cultural and linguistic nuances. These strategies can mitigate mistrust, reduce mental health stigma, and enhance service engagement.

Researchers may prioritise longitudinal studies examining how structural racism, poverty, and educational inequities cumulatively affect Black youth across the life course. Intersectional analyses are urgently needed to elucidate how overlapping identities (e.g., race, gender, immigration status) compound health risks, particularly for marginalised subgroups such as Black girls in low-income households. Geographic disparities also demand attention, as provinces like Saskatchewan, Newfoundland and Labrador, and Prince Edward Island remain understudied. Disaggregating data by ethnicity, gender, age, and migration status may clarify heterogeneity within Black communities, moving beyond reductive “visible minority” classifications.

## Conclusion

This scoping review provided a critical overview of the health of Black (African and Caribbean) children and youth in Canada. Our findings revealed high disease burden and underutilisation of healthcare services, largely driven by systemic racism and SDOH. These findings underscore the urgent need for targeted policy and healthcare interventions. Specifically, we recommend implementing anti-racism frameworks across healthcare and social systems; investing in culturally responsive and community-led services; increasing the recruitment and support of Black health professionals; and integrating culturally safe care. Furthermore, researchers may prioritise intersectional and longitudinal approaches to address the unique health needs of Black children and youth across Canada. These recommended strategies may advance health equity and improve the health outcomes for this population.

## Supplementary Information

Below is the link to the electronic supplementary material.

Supplementary Material 1

Supplementary Material 2

Supplementary Material 3

## Data Availability

The data is in supplementary file 3.

## Abbreviations

AIDS: Acquired Immunodeficiency Syndrome
APA: American Psychological Association
COVID-19: Coronavirus Disease 2019
EMBASE: Excerpta Medica Database
HIV: Human Immunodeficiency Virus
MEDLINE: Medical Literature Analysis and Retrieval System Online
MeSH: Medical Subject Headings
PTSD: Post-Traumatic Stress Disorder
PRISMA-ScR: Preferred Reporting Items for Systematic Reviews and Meta-Analyses extension for Scoping Reviews
